# Evidence of a Synergistic Effect of Acupoint Combination: A Resting-State Functional Magnetic Resonance Imaging Study

**DOI:** 10.1089/acm.2016.0016

**Published:** 2016-10-01

**Authors:** Jiping Zhang, Yu Zheng, Yanjie Wang, Shanshan Qu, Shaoqun Zhang, Chunxiao Wu, Junqi Chen, Huailiang Ouyang, Chunzhi Tang, Yong Huang

**Affiliations:** ^1^ School of Traditional Chinese Medicine, Southern Medical University, Guangzhou, China.; ^2^ Department of Rehabilitation Medicine, Third Affiliated Hospital of Southern Medical University, Guangzhou, China.; ^3^ Department of Traditional Chinese Medicine, Zhujiang Hospital of Southern Medical University, Guangdong, China.; ^4^ Clinical Medical College of Acupuncture, Moxibustion and Rehabilitation, Guangzhou University of Chinese Medicine, Guangzhou, China.

**Keywords:** acupuncture, acupoints combination, MRI, synergistic effect

## Abstract

***Objective:*** This study aimed to find evidence of a synergistic effect of acupoint combinations by analyzing different brain regions activated after acupuncture at different acupoint combinations.

***Methods:*** A total of 57 healthy subjects were randomly distributed into three groups: LR3 plus KI3 acupoints, LR3 plus sham acupoint, or LR3 alone. They underwent a magnetic resonance imaging scan before and after acupuncture. The amplitude of low-frequency fluctuation (ALFF) and regional homogeneity (ReHo) values of different brain regions were analyzed to observe changes in brain function.

***Results:*** ALFF and ReHo produced an activated area in the cerebellum posterior lobe after acupuncture at LR3 plus KI3 acupoints versus LR3 alone. ALFF and ReHo revealed altered activity in Brodmann area 10 (BA10), BA18, and brainstem pons after acupuncture at LR3 plus sham acupoint compared with at LR3 alone. A comparison of acupuncture at LR3 plus KI3 acupoints with LR3 plus sham acupoint demonstrated an increase in BA6 of ALFF and a downregulation of ReHo.

***Conclusions:*** The increased number of brain regions with altered brain activity after acupuncture at acupoint combinations versus a single acupoint are evidence of the synergistic effect of acupoint combinations. BA6 was significantly activated after acupuncture at LR3 plus KI3 acupoints compared with at LR3 plus sham acupoint, suggesting that BA6 is the specific region of synergistic effect of acupoint combinations of LR3 plus KI3 acupoints. Affected brain regions were different between acupuncture at LR3 plus sham acupoint and LR3 alone, which indicates that the sham acupoint may have some psychological effect. However, the specific mechanism of acupoint combinations requires further research.

## Introduction

Acupuncture is an important part of Traditional Chinese Medicine, and it is widely applied for the treatment of various diseases, such as motor-system and nervous-system diseases. Many randomized controlled trials and evidence-based medicine have fully affirmed the remarkable curative effect of acupuncture on a variety of diseases.^1–3^

Acupoint combinations account for the vast majority of prescribed acupuncture points. Acupoint combination means the use of two or more acupoints at once during acupuncture therapy, and its action can be classified into synergy, antagonism, or other effects (e.g., additive, no interaction, and mixed). Synergy occurs when the effect of an acupoint combination is stronger than the simple sum of the effects of individual acupoints, whereas antagonism means that the effect of acupoint combinations is weaker than the sum of the effects of individual acupoints using a variety of indicators.^4^ Undoubtedly, synergy of acupoint combinations is a desirable therapeutic effect.

Clinical trials have shown significantly better efficacy of acupoint combinations compared with a single acupoint.^5–7^ Animal experiments demonstrated the efficacy of acupuncture using acupoint combinations at the molecular level. For example, Pan et al.^8^ reported that electroacupuncture (EA) at feishu (BL13) and xinshu (BL15) acupoints protected against pulmonary hypertension by regulating the activity of endothelium-derived endothelin 1 (ET-1) and endothelial nitric oxide synthase (eNOS). Yan et al.^9^ found that EA of the Zhongwan (CV12), Tianshu (ST25), or Shangjuxu (ST37) acupoints alone or in combination relieved acetic acid–induced intestinal mucosal lesions in rats, and the effect of joint application of these three acupoints was significantly better. Nevertheless, the mechanisms of synergy have not been elucidated.

The effect of acupoint combinations can be objectively assessed using measurements of functional brain activity.^10^ Brain regions respond to acupuncture in different acupoint combinations with different activity signals, which are collected, compared, processed, and delivered to target organs. These data demonstrate the compatibility of different acupoint combinations based on various clinical effects.

Currently, functional brain-imaging research focuses on the specificity of meridians and acupoints. Previous studies using functional magnetic resonance imaging (fMRI) compared differences in brain function between acupuncture at a single acupoint and the surrounding (nonacupoint) area^11–13^ or different acupoints.^14–17^ These studies confirmed the specificity of meridians and acupoints in relation to brain function. However, these studies evaluated changes in brain function as a result of a single acupoint, and few previous studies investigated the effects of acupoint combinations. Huang et al.^18^ examined fMRI in healthy people receiving acupuncture at the Waiguan (SJ5) versus Waiguan plus Yanglingquan (GB34) acupoints. They found the acupuncture point combination of SJ5 and GB34 within the hand–foot Shaoyang meridians improved motor and sensory dysfunction and equilibrium disturbances. Nevertheless, these acupuncture studies were based on a block design, that is, stimulation using an “acupuncture-resting-acupuncture-resting” scheme, which is not clinically suitable. The present study tested acupuncture in different acupoint combinations using resting-state fMRI, and compared the imaging data to verify changes in brain functional connectivity in different brain regions.

Previous studies demonstrated persistent effects of acupuncture. Vitally, Napadow et al.^19^ observed a linearly decreasing time variation in the activation of sensorimotor brain regions in response to electrostimulation at the acupoint Zusanli (ST36) and a sham point, which suggested classical habituation. Zheng et al.^20^ found that changes in number and intensity were greater at 15 min after EA stimulation at Yintang and Baihui (GV20) compared to 5 min after needle removal, which demonstrated lasting and strong aftereffects of EA on functional cerebral regions.

The LR3 acupoint has been widely used to study the specificity of meridians and acupoints. Wu et al.^21^ and Li et al.^22^ certified the specificity of acupoints. This study used LR3 as a test acupoint to identify possible differences in functional brain changes between acupuncture at LR3 plus KI3 acupoints and LR3 alone on the basis of the previous studies to explain the mechanism of action of acupoint combinations. In addition, since it currently not clear whether all acupoint combinations have synergistic effects, this study also assessed brain activity after acupuncture at LR3 plus a sham acupoint and compared it with the response to acupuncture at LR3 plus KI3 and LR3 alone to identify more evidence of the synergistic effects of acupoint combinations.

The amplitude of low-frequency fluctuation (ALFF) and regional homogeneity (ReHo) were used in this study to measure brain function. ALFF represents the intensity of a blood oxygen level–dependent signal in each voxel, which directly reflects the spontaneous activity of neurons via energy expenditure. ReHo primarily reflects the synchronism of a time series in regional brain areas, not signal intensity, and it indirectly reflects the synchronism of the spontaneous activity of local neurons in a specific brain region.^23^ Therefore, this study evaluated the specificity of LR3 by analyzing alterations in regional brain activities using ReHo and ALFF. The results of two analytical methods were organized and compared with the observed effects of acupuncture on cerebral functional activities to draw more accurate and comprehensive conclusions.

In summary, this study assessed the affected brain regions after acupuncture at different acupoint combinations (LR3 plus KI3 acupoints, LR3 plus sham acupoint, or LR3 alone) using MRI. Changes in brain functional were compared to evaluate possible evidence of synergistic effects of acupoint combinations.

## Materials and Methods

### Participants

Based on the previous report about minimum sample size in neuroimaging studies,^24^ a sample size of 16 per group was needed (total *N* = 48). Considering a conservative dropout rate of 15%, a total sample size of 57 subjects was determined.

Participants were recruited between September 5, 2012, and January 14, 2013. All of the participants consented to participate in this study and for the case details to be published. Written informed consent was obtained from all participants involved in this study.

**FIG. 1.**
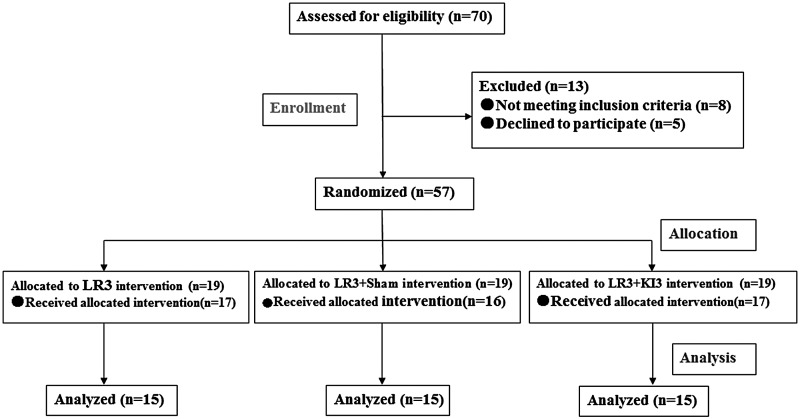
Consort flowchart.

Participants were healthy young people from universities and colleges in Guangzhou City, China. The following inclusion criteria were used: aged between 21 and 28 years; never received acupuncture; a regular diet; minimal consumption of liquor, tobacco, tea, and coffee; normal sleep patterns (e.g., going to bed before midnight); moderate weight (body mass index 18.5–23.9 kg/m^2^); no pain (including dysmenorrhea) or insomnia within a month before the test; and no damaged skin around the test acupoints. All subjects passed a pilot acupuncture test that was performed one month before the study, and all subjects gave full informed consent (Fig. 1)

### Random allocation

This randomized, controlled, single-blind trial was conducted at the First Affiliated Hospital of Guangzhou University of Chinese Medicine. According to complete randomized block design, participants were randomly distributed into three groups: LR3 plus KI3 acupoints, LR3 plus a sham acupoint, or LR3 alone. An independent biostatistics professionals generated the random allocation sequence using IBM SPSS Statistics for Windows v19 (IBM Corp., Armonk, NY) and put the random number into opaque sealed envelopes. An investigator who did not participate in the acupuncture interventions controlled the sealed envelopes. Investigators who selected the eligible participants after baseline screening opened the envelopes according to the patients’ screening sequence numbers, enrolled participants, and assigned participants to receive interventions.

### Blinding

Due to the procedure of the acupuncture technique, the acupuncture physician in this study was not blinded. Investigators in charge of patient screening and randomized distribution were not involved in intervention and data analyses. The subjects were blinded to the acupoints where they received acupuncture. The acupuncture intervention was performed in a large independent single room with screen dividers for patient blinding and privacy, and subjects’ eyes were covered with eyeshades during acupuncture intervention. The statistician, in charge of statistical analysis and independent of acupuncture intervention and data assessor, was blinded to the randomization allocation.

### Intervention

**FIG. 2.**
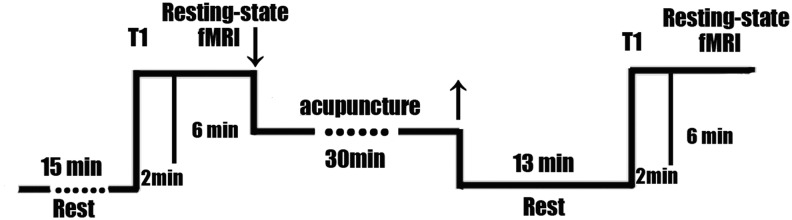
Trial flowchart.

Subjects were asked to pass urine and stool prior to the intervention. Subjects rested for 15 min at the beginning of the experiment and then underwent the MRI scan and acupuncture. The timeline of acupuncture stimulation and MRI scan is shown in Figure 2.

### Acupuncture stimulation

The same experienced acupuncture physician, who received special training prior to the study to ensure consistent manual acupuncture therapy, performed acupuncture on all subjects. Each subject received acupuncture only once in one of the three combinations: LR3 alone, LR3 plus sham, or LR3 plus KI3. Subjects’ eyes were covered with eyeshades so that subjects were blinded to the acupoints where they received acupuncture.

### Acupuncture localization

**FIG. 3.**
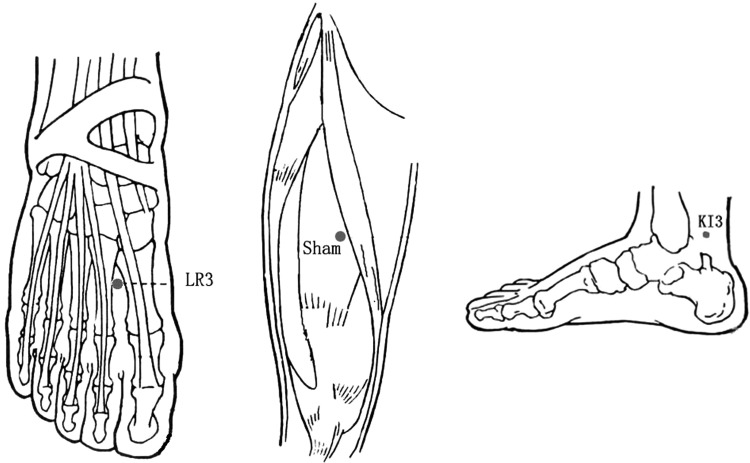
Location of acupoints.

Acupoints were localized according to the name and location of acupoints (Chinese National Standards GB/T12346; Fig. 3). These were: LR3—on the dorsum of the foot, in the depression anterior to the junction of the first and second metatarsal bones; KI3—in the depression between the tip of the media malleolus and tendo calcaneus; and sham point—on the midpoint of the line connecting the anterior superior iliac spine and lateral border of the patella, 2 cm inside.

### Acupuncture operation

The physician's hands and subjects’ skin around the acupoints were sterilized with alcohol before needling. Huatuo needles (0.30 × [25–45 mm]; Suzhou Medical Supplies Co., Suzhou, China) were used in this study.

Acupuncture was performed using fingernail press insertion. The order of acupoints was right LR3 → left LR3 → right KI3/sham point → left KI3/sham point. The skin was vertically punctured to a depth of 15 ± 2 mm. Needles were twirled at an angle of 90–180° and a frequency of 60–90 times/min after a participant sensed the needle. Needles were lifted and thrust in the range of 0.3–0.5 cm and frequency of 60–90 times/min. Needles were manipulated for 1 min and held in place for 30 min. The physician manipulated the needle for 1 min every 10 min during this 30 min.

### fMRI examination

The fMRI scanning was carried out in a 3.0 Tesla Signa HDxt MRI scanner (GE Company, Fairfield, CT) at First Affiliated Hospital of Guangzhou University of Chinese Medicine. A standard eight-channel phase-array head coil and restraining foam pads were used to minimize head motion.

Subjects were conscious, placed in a supine position, and asked to breathe calmly. Earplugs and a special earshield were used to diminish scanner noise, and eyeshades were used to avoid visual stimulation. During the fMRI scanning, subjects were instructed to move as little as possible and, if they felt uncomfortable, to tell investigators loudly and the scan would be stopped. fMRI scanning began after subjects rested for 15 min.

MRI data (resting-state BOLD sequence) were collected 15 min before needling and 15 min after needle withdrawal. Scanning methods were identical between sham and true acupuncture.

(1) Transverse T1-weighted image (T1WI) sequence: 1 min 51 sec, fast spin echo sequence; OAx T1 FLAIR, repetition time 1750 ms/echo time 24 ms, inversion time 960 ms, field of view 24 cm × 24 cm/Z, matrix 320 × 224/number of excitations = 1, thickness 5.0 mm/interval 1.0 mm, 30 slices total, echo train length 8, and bandwidth 31.25.

(2) Resting-state fMRI BOLD data collection: gradient echo-echo-planar imaging sequence scanning was conducted for 6 min in accordance with the following parameters: repetition time 3000 ms/minimum, echo time minimum, flip angle 90°, field of view 240 mm × 240 mm, thickness 5.0 mm/interval 1.0 mm, 30 slices each time, matrix 96 × 96/ number of excitations = 1.

### Image preprocessing and analytical methods

Preprocessing was performed using Data Processing Assistant for Resting-State fMRI (DPARSF v2.3; http://rfmri.org/DPARSF),^41^ which is based on Statistical Parametric Mapping (SPM8; www.fil.ion.ucl.ac.uk/spm) and a Resting-State fMRI Data Analysis Toolkit (REST 1.8; www.restfMRI.net).^26^

The preprocessing procedure includes: (1) convert DICOM to NIFTI; (2) slice timing after removing first 10 time points; (3) realign and exclude subjects with max head motion >1.5 mm on any axis and head rotation >1.5°; (4) co-register T1 to Fun; (5) segment and affixer regularization according to East Asian; (6) normalize by using EPI templates; (7) smooth images with a Gaussian kernel with a isotropic full-width at half-maximum (FWHM) of 4 mm (data for ReHo analysis without smooth); (8) remove linear detrend; and (9) filter (0.01–0.08 Hz).

#### ReHo analysis

ReHo was calculated by REST software. The Kendall's coefficient of concordance (KCC) of each voxel was calculated by the time series of the voxel and its nearest 26 neighboring voxels (cluster size = 27). Then the KCC maps were standardized by dividing their own mean KCC within the whole brain mask, and the resulting maps were smoothed with a Gaussian kernel with 4 mm FWHM.^23,25,26^

#### ALFF analysis

ALFF was calculated by REST software. The time series of each voxel was converted to the frequency domain using a fast Fourier transform. Then the square root of the power spectrum was computed and averaged across a predefined frequency interval. The average square root was termed ALFF.^27^ Next, the ALFF was standardized by dividing the mean ALFF within the whole brain mask.

### Statistical analysis

Data were analyzed using REST1.8 software. In the statistical analysis, one-way analysis of variance (ANOVA) was used to explore standardized ALFF/ReHo value differences among the three groups with AlphaSim correction 5 and continuous voxel >85. To illustrate clearly the difference between groups, the comparison was further performed on ALFF/ReHo maps using a two-sample *t*-test with Bonfferoni correction (*p* < 0.0167) and continuous voxel >85. Finally, ALFF/ReHo value alteration differences among different groups were obtained. Rest1.8 software Viewer was employed to identify the precise anatomical position in the brain with statistical significance on the corresponding MNI coordinate. The results are presented as images.

## Results

### Participants

Fifty-seven subjects were recruited from the Southern Medicine University and Guangzhou University of Chinese Medicine, China. They were equally allocated into three groups: LR3 alone, LR3 plus sham point, and LR3 plus KI3 acupoints (19 in each group). Two subjects from the LR3 alone group, three subjects from the LR3 plus sham point group and two subjects from the LR3 plus KI3 acupoints group dropped out during the study for the following reasons: could not bear the MRI scanner noise (*n* = 2); noncompliance with study schedule (*n* = 1); and sudden power outages as MRI imaging data acquiring (*n* = 4). Group images from two subjects from the LR3 alone group, three subjects from the LR3 plus sham point group, and two subjects from the LR3 plus KI3 acupoints were excluded during data preprocessing, as max head motion was >1.5 mm on any axis and head rotation was >1.5°. Finally, the fifth subject in each group of images was included for statistical analysis. The baseline and demographics of age, sex, height, and weight with the ITT population are shown in Table 1, which showed that the three groups were comparable at baseline. Furthermore, there was no significant difference between groups in functional imaging before acupuncture according to the REST software.

**Table 1.** Participants’ Baseline Data

		*Sex*				
*Group*	n	*Male*	*female*	*Age, years*	*Height, cm*	*Weight, kg*
LR3	15	9	6	21.67 ± 0.488	168.60 ± 6.811	55.40 ± 8.348
LR3 plus sham	15	8	7	21.47 ± 0.516	163.80 ± 7.747	53.87 ± 6.967
LR3 plus KI3	15	7	8	21.47 ± 0.516	166.13 ± 7.482	55.87 ± 7.586

### Brain regions showing changes after acupuncture at the acupoints LR3 plus KI3 versus LR3 alone

#### ALFF analysis

**FIG. 4.**
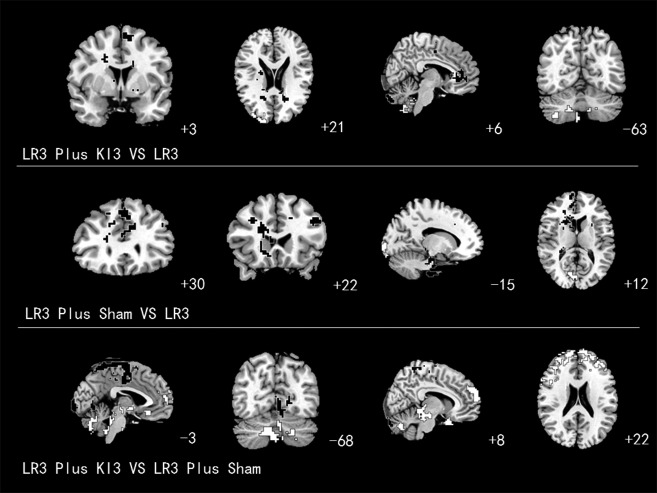
Brain areas with amplitude of low-frequency fluctuation alteration. *White* areas with *black line* represent deactivation; *black* areas with *white line* represent activation.

Increased ALFF (T value was positive) was detected in the left superior frontal gyrus (Brodmann area 6 [BA6]), right frontal lobe subgyral corpus callosum (BA32), and subgyral right frontal lobe (BA24). Decreased ALFF (T value was negative) was observed in the right middle frontal gyrus (BA10), right cerebellum posterior lobe pyramis, right cerebellum posterior lobe, and right inferior semilunar lobule (Table 2 and Fig. 4).

**Table 2.** Brain Areas with ALFF Alteration

					*Talairach (mm)*			
*Comparisons*	*Number of voxels*	*Brain areas*	*Right/left*	*Brodmann area*	*X*	*Y*	*Z*	*T*
LR3 plus KI3 vs. LR3	122	Superior frontal gyrus	L	6	−3	3	63	4.7498
	849	Frontal lobe, sub-gyral, corpus callosum	R	32	15	24	21	4.6052
	85	Frontal lobe, sub-gyral	R	24	21	3	36	3.7494
	148	Middle frontal gyrus	R	10	42	54	12	−3.6715
	138	Cerebellum, posterior lobe, pyramis	R		6	−90	−33	3.8422
	248	Cerebellum posterior lobe, inferior semi-lunar lobule	R		36	−63	−54	−3.5093
LR3 plus sham vs. LR3	135	Superior frontal gyrus	L	8	0	30	48	3.974
	93	Middle frontal gyrus	L	9	−51	21	33	3.9274
	121	Occipital lobe, cuneus	L	18	−15	−99	0	−3.4428
	282	Left brainstem, pons	L		−15	−21	−27	4.2434
	361	Frontal lobe, frontal forceps	R	9	18	24	21	4.9586
	118	Superior frontal gyrus	R	10	9	63	12	3.9919
	103	Sub-lobar, extra-nuclear	R	37	27	−36	12	3.7882
	153	Occipital lobe, cuneus	R	18	12	−78	12	−4.8673
LR3 plus KI3 vs. LR3 plus sham	575	Frontal lobe, paracentral lobule	L	6	−3	−33	69	5.0682
	300	Occipital lobe, cuneus	L	18	−3	−102	−3	4.07
	113	Limbic lobe, posterior cingulate	L	18	18	−66	3	−4.8673
	1196	Inferior frontal gyrus	R	10, 9	48	9	22	−6.9094
	261	Cerebellum posterior lobe, cerebelum_Crus2_R	R		9	−93	−30	4.618
	201	Cerebellum posterior lobe, uvula	R		15	−69	−42	−3.8729
	770	Right brainstem, midbrain	R		3	−33	−18	−5.2822

ALFF, amplitude of low-frequency fluctuation.

#### ReHo analysis

**FIG. 5.**
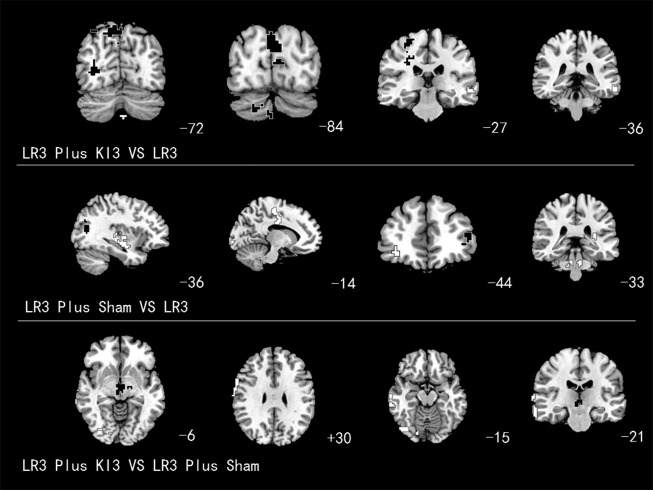
Brain areas with regional homogeneity alteration. *White* areas with *black line* represent deactivation; *black* areas with *white line* represent activation.

Increased ReHo was detected in the right superior parietal lobule (BA7), right frontal lobe precentral gyrus (BA4), right temporal subgyral lobe (BA22), right cerebellum posterior lobe, left occipital lobe, and cuneus (BA19, 18). Decreased ReHo was observed in the left middle temporal gyrus (BA21; Table 3 and Fig. 5).

**Table 3.** Brain Areas with ReHo Alterations

					*Talairach (mm)*			
*Comparisons*	*Number of voxels*	*Brain areas*	*Right/left*	*Brodmann area*	*X*	*Y*	*Z*	*T*
LR3 plus KI3 vs. LR3	106	Superior parietal lobule	R	7	15	−72	51	4.6556
	146	Frontal lobe, precentral gyrus	R	4	27	−27	66	4.5514
	89	Temporal lobe, sub-gyral	R	22	36	−63	6	4.1554
	86	Cerebellum posterior lobe	R		9	−87	−39	3.9505
	138	Occipital lobe, cuneus	L	19, 18	−3	−84	24	4.4965
	93	Middle temporal gyrus	L	21	−66	−36	−18	−4.0062
LR3 plus sham vs. LR3	91	Superior occipital gyrus	L	39, 19	−33	−75	24	4.7224
	87	Inferior frontal gyrus	L	46	−48	42	9	3.7627
	161	Superior temporal gyrus	L	22	−36	−12	−15	−4.7235
	103	Medial frontal gyrus	L	24, 6	−12	−21	57	−4.4383
	96	Occipital lobe, cuneus	L	18	−18	−99	−3	−4.0951
	86	Middle frontal gyrus	R	10	36	48	−9	−5.0098
	88	Right brainstem, pons	R		6	−33	−33	−3.5016
LR3 plus KI3 vs. LR3 plus sham	85	Sub-lobar, extra-nuclear	R		3	−12	−6	4.1944
	94	Frontal lobe, precentral gyrus	R	6	66	−3	30	−4.3068
	130	Occipital lobe, lingual gyrus	R	19	18	−84	−15	−4.0172
	110	Middle temporal gyrus	R	21	72	−21	3	−3.8863

ReHo, regional homogeneity.

### Brain regions with changes after acupuncture at the LR3 plus sham acupoints versus LR3 alone

#### ALFF analysis

ALFF apparently increased in the left superior frontal gyrus (BA8), left middle frontal gyrus (BA9), left brainstem pons, right frontal forceps (BA9), right superior frontal gyrus (BA10), and right sublobar extranuclear (BA37) and decreased in the left and right occipital lobe cuneus (BA18; Table 2 and Fig. 4).

#### ReHo analysis

Increased ReHo was detected in the left superior occipital gyrus (BA39 and BA19) and left inferior frontal gyrus (BA46). Decreased ReHo was observed in the left superior temporal gyrus (BA22), left medial frontal gyrus (BA24 and BA6), left occipital lobe cuneus (BA18), right middle frontal gyrus (BA10), and right brainstem pons (Table 3 and Fig. 5).

### Brain regions with changes after acupuncture at the LR3 plus KI3 acupoints versus LR3 plus sham

#### ALFF analysis

Increased ALFF was detected in the left frontal lobe paracentral lobule (BA6), left occipital lobe cuneus (BA18), and right cerebellum posterior lobe cerebelum_crus2_R. Decreased ALFF was observed in the left limbic posterior cingulate lobe (BA18), right inferior frontal gyrus (BA10 and BA9), right cerebellum posterior lobe uvula, and right brainstem midbrain (Table 2 and Fig. 4).

#### ReHo analysis

Increased ReHo was detected in the right sublobar extranuclear region. Decreased ReHo was observed in the right frontal lobe precentral gyrus (BA6), right occipital lobe lingual gyrus (BA19), and right middle temporal gyrus(BA21; Table 3 and Fig. 5).

## Discussion

Comparison of LR3 plus KI3 acupuncture versus LR3 alone revealed that ALFF alterations were concentrated in BA6, BA10, BA24, BA32, the cerebellum posterior lobe, and inferior semilunar lobule regions of the brain. ReHo alterations were observed in BA4, BA7, BA18, BA19, BA21, BA22, and cerebellum posterior lobe regions of the brain. The ALFF and ReHo results demonstrated that cerebellum posterior lobe regions were activated brains areas. The simultaneously increased values of ALFF and ReHo demonstrated that the local neuronal activity of cerebellum posterior lobe regions was enhanced, and the surrounding neuronal activity was coordinated more closely. The cerebellum posterior lobe maintains body balance, participates in the initial processing of sensory and emotional information, and regulates neurological function.^28^ The activity of the posterior lobe of the cerebellum has a modulatory effect in the treatment of ventricular hypertrophy,^29^ which suggests that acupuncture at the acupoints LR3 plus KI3 strengthens the function of the cerebellar posterior lobe. Movement disorders often involve impaired functioning of the cerebellum, which can be hypo-or hyperactive compared with healthy functioning.^30^ So the increased cerebellum common activity indicates acupuncture at LR3 plus KI3 acupoint combination can be used to treat movement diseases such as tremor, dystonia, and so on.

Previous studies found that acupuncture at LR3 specifically activates BA7, BA18, BA19, and other brain regions.^22,31^ BA7, BA18, and BA19 are involved in the formation of visual transduction, and one study showed that BA7 and the posterior cingulate were the information-processing hubs that connect the dorsal cochlear nucleus and ventral palladium.^32^ In addition, stimulation of LR3 also activated the thalamus and the limbic system. These areas were involved in visceral modulation.^15^ Therefore, acupuncture at LR3 could be applied to the treatment of vision-related diseases as well as visceral pain and body paralysis. Chen et al. found that acupuncture at KI3 enhanced active connections of the post-temporal cortex, dorsolateral prefrontal cortex, and the ventromedial prefrontal cortex.^33^ Zhong et al.^34^ found that acupuncture at KI3 increased incoming and outgoing activity of local superior temporal gyrus neurons and strengthened the connection of the superior temporal gyrus with the posterior central gyrus.

The present study found brain regions associated with visual and auditory perception, body movement, and association are selectively activated after acupuncture at KI3. Acupuncture at KI3 acts on certain brain regions related to its clinical application, as acupuncture at KI3 is usually used in the treatment of developmental retardation, presenility, diseases of the urogenital system, headache, auditory disorders, cognitive dysfunctions, insomnia, chronic cough, and chronic diarrhea, according to Traditional Chinese Medicine.^35^ The post-temporal cortex and superior temporal gyrus are closely related to the formation of auditory sense. Therefore, KI3 is widely used in the treatment of hearing-related diseases. The functional activities of visual center–related brain areas (BA7, BA18, BA19, and BA41) and auditory event–related brain regions (BA21 and BA22) are activated with the combination of LR3 and KI3, which increases the related neuronal spontaneous activity levels of vision and hearing. Therefore, the LR3 and KI3 combination could be more beneficial than the single acupoint in the treatment of audiovisual diseases. The increased number of activated brain regions confirms that the combining of acupoints has a synergistic effect on the central nervous system. Huang et al.^18^ and Leung et al.^36^ also confirmed that combining acupoints produces a synergistic effect using functional brain imaging techniques.

Acupuncture at LR3 plus sham produced ALFF alterations in BA8, BA9, BA10, BA18, BA37, and brainstem pons regions. ReHo produced affects in the BA6, BA10, BA18, BA19, BA22, BA24, BA46, and brainstem pons regions. ALFF and ReHo both showed that the common affected areas were BA10, BA18, and brainstem pons regions. BA10 is located in the prefrontal lobe, and it participates in association and management function. A previous study showed that BA10 strengthened the functional connectivity of amygdaloidal nucleus, anterior cingulate, and superior frontal gyrus cortex when subjected to continuous pressure.^37^ Another study found that the ALFF value of BA10 was significantly higher in patients with post-traumatic stress disorder.^38^ BA10 ALFF value increased and ReHo values decreased as a result of the combination of the LR3 plus sham acupoints, which suggests that the local neuronal activity of BA10 increased, but the surrounding neuronal activity is not consistent with the changes in BA10. These data indicate that this effect may be the result of differences in the positioning and *de qi* sensation of the nonacupuncture point and the true acupoint, which caused subjects to generate relevant associations. Therefore, the functional activity changes in BA10 may be the result of the sham acupoint's influence on the acupoint. In contrast, the ALFF value of BA10 decreased in the combination KI3 plus LR3. Both acupoint combinations affected the functional activities of BA10, but the roles of the results were different, which may also be the crucial evidence of synergy of this acupoint combination. A lower value of ALFF and ReHo means that the relative signal of local oxygen was reduced, which breaks the connection with surrounding neurons. These results may be a sign of neuronal inhibition.^39^ Inhibition of BA18 means that vision-related neuronal activity levels decrease as a result of the LR3 plus sham combination. These data are consistent with a previous study.^40^ ReHo values of BA18 decreased as a result of the LR3 plus sham combination, whereas the ReHo value increased under the influence of the LR3 plus KI3 combination. This phenomenon may be the central difference between the combinations LR3 plus KI3 and LR3 plus sham. However, it may be a normal effect of adjustment (excitatory and inhibitory). It is possible that acupoint combinations have novel central effects, and nonacupoints (sham) impact LR3, which does not correspond to a 1 + 0 = 1 relationship. The sham acupoint impacts the central mechanisms of LR3, but their specific interactions require further in-depth research.

Comparisons of acupuncture at the LR3 plus KI3 acupoints to the LR3 plus sham revealed that ALFF alterations were concentrated in BA6, BA9, BA10, BA18, and the brainstem midbrain, cerebellum crus, and cerebellar posterior lobe regions of the brain. ReHo alterations were observed in BA6, BA19, BA21, and the sublobar and extranuclear regions of the brain. The ALFF and ReHo results demonstrated that the common activated brain region was BA6, which exhibited increases in ALFF and decreases in ReHo. Enhanced ALFF means an increase in regional blood oxygen signal intensity, which is associated with more local field potentials and multiunit activities. A smaller value of ReHo indicates lower regional synchronization. Synchronization reflects intrinsic coherent neuronal activity within spatially organized brain regions, which may be relevant to neural inactivity in a regional brain area.^23,26,41^ BA6 is a motor cortical area in the posterior frontal lobe that is directly anterior to the primary motor cortex. This area participates in the planning and execution of volitional movement. BA6 receives significantly higher regional cerebral blood flow across pain modalities.^42^ Therefore, it was hypothesized that acupuncture at the LR3 plus KI3 acupoints have a synergistic effect on pain. Acupuncture at the LR3 plus KI3 acupoints is widely used to regulate blood pressure. Cell et al.^43^ showed that high blood-pressure levels were associated with a smaller gray-matter volume in the supplementary motor area (BA6). Another study suggested that changes in the infrared radiation temperature of the lower extremity acupoints KI3 and LR3 are closely related to the pathological characteristics of the dysfunction of conception and thoroughfare vessels syndrome of hyperplasia of the mammary gland.^44^ Therefore, changes in BA6 function after acupuncture at the LR3 plus KI3 acupoints are evidence of a synergistic effect of this acupoint combination.

Furthermore, BA18, BA19, BA21, and visual association areas were inactivated, which are signs of visual neuronal inhibition. Previous studies confirmed that acupuncture at LR3 induced specific patterns of activities in visual association brain areas.^18,25^ Common activated areas were found in response to LR3 plus KI3 or LR3 plus sham in visual association brain areas. Therefore, this phenomenon represents a synergistic effect of this acupoint combination, which is an extension of the individual acupoint effects.

This study has some limitations. All subjects in this study were healthy. Previous research demonstrated that signal activation patterns during the same acupuncture procedure are different between healthy subjects and ill patients.^21,45^ Accordingly, the results could only be tentatively presumed useful for the treatment of relevant diseases. A further study will assess the mechanisms of acupoints combinations in pathological conditions.

## Conclusion

The increased number of altered brain regions as a result of acupuncture at the LR3 plus KI3 acupoints versus LR3 alone supports a synergistic effect of acupoint combinations. BA6 was activated more strongly after acupuncture at the LR3 plus KI3 acupoints compared with LR3 plus sham, which suggests that BA6 is one of the specific brain regions for the LR3 plus KI3 combination. Some additional alterations were observed in brain regions after acupuncture at LR3 plus sham compared with acupuncture at the LR3 acupoint alone. These data suggest that changes in brain activity after acupuncture at LR3 are affected by the sham acupoint, but the mechanisms of their interaction need further research.
